# Does Consumer Credit Precede or Follow Health Among Older Adults? An Investigation in the Advanced Cognitive Training for Independent and Vital Elderly (ACTIVE) Trial

**DOI:** 10.1093/geroni/igae016

**Published:** 2024-02-22

**Authors:** Lorraine T Dean, Shang-En Chung, Alden L Gross, Olivio J Clay, Sherry L Willis, Ian M McDonough, Kelsey R Thomas, Michael Marsiske, Jaya Aysola, Roland J Thorpe, Cynthia Felix, Melissa Berkowitz, Norma B Coe

**Affiliations:** Department of Epidemiology, Johns Hopkins Bloomberg School of Public Health, Baltimore, Maryland, USA; Department of Epidemiology, Johns Hopkins Bloomberg School of Public Health, Baltimore, Maryland, USA; Department of Epidemiology, Johns Hopkins Bloomberg School of Public Health, Baltimore, Maryland, USA; Department of Psychology, University of Alabama at Birmingham, Birmingham, Alabama, USA; University of Alabama at Birmingham Alzheimer’s Disease Research Center, Birmingham, Alabama, USA; Department of Psychiatry and Behavioral Sciences, University of Washington, Seattle, Washington, USA; Department of Psychology, University of Alabama, Tuscaloosa, Alabama, USA; VA San Diego Healthcare System and Department of Psychiatry, University of California, San Diego School of Medicine, San Diego, California, USA; Department of Clinical and Health Psychology, University of Florida, Gainesville, Florida, USA; Department of Medicine and Pediatrics, Perelman School of Medicine, University of Pennsylvania, Philadelphia, Pennsylvania, USA; Department of Health, Behavior and Society, Johns Hopkins Bloomberg School of Public Health, Baltimore, Maryland, USA; Johns Hopkins Alzheimer’s Disease Resource Center for Minority Aging Research, Baltimore, Maryland, USA; Department of Epidemiology, University of Pittsburgh, Pittsburgh, Pennsylvania, USA; Department of Medical Ethics and Policy, Perelman School of Medicine, University of Pennsylvania, Philadelphia, Pennsylvania, USA; Department of Medical Ethics and Policy, Perelman School of Medicine, University of Pennsylvania, Philadelphia, Pennsylvania, USA

**Keywords:** Attitude to health, Financial activities, Patient credit

## Abstract

**Background and Objectives:**

Consumer credit has shown increasing relevance to the health of older adults; however, studies have not been able to assess the extent to which creditworthiness influences future health or health influences future creditworthiness. We assessed the relationships between 4-year pre and postmorbid consumer credit history and self-rated physical and mental health outcomes among older adults.

**Research Design and Methods:**

Generalized estimating equations models assessed pre and postmorbid credit history (credit scores, derogatory accounts, and unpaid accounts in collections) and the onset of poor self-rated health (SF-36 score <50) among 1,740 participants aged 65+ in the Advanced Cognitive Training for Independent and Vital Elderly study from 2001 to 2017, linked to TransUnion consumer credit data.

**Results:**

In any given year, up to 1/4 of participants had a major derogatory, unpaid, or collections account, and up to 13% of the sample had poor health. Each 50-point increase in credit score trended toward a 5% lower odds of poor health in the next 1 year, a 6% lower odds in the next 2 years, and a statistically significant finding of 13% lower odds by 3 years. A drop in credit score was associated with a 10% greater odds of poor health in the next year, and having a major derogatory account was associated with an 86% greater odds of poor health in the next 3 years. After poor health onset, credit scores continued to see significant losses up to the 3 years, with larger decrements over time.

**Discussion and Implications:**

Having a major derogatory account or a sudden loss in credit may be a time to monitor older adults for changes in health. After a downturn in health, supporting older adults to manage their debt may help stabilize their credit.

**Translational Significance:** An emerging body of evidence links consumer credit to health; however, studies have not disentangled the temporal ordering of credit with general physical or mental health for older adults. Our results suggest that credit may signal a downturn in older adult health up to 3 years before it happens and that a downturn in health impacts credit for up to 3 years later, with more pronounced associations with mental than physical health. Monitoring credit could help with early identification of emerging poor health in older adults, or determine when to intervene to stabilize credit after poor health onset.

## Background and Objectives

An emerging body of research suggests that better consumer credit is associated with better general health and well-being or fewer self-reported diseases (Bernerth et al., 2012; Brockett & Golden, 2007; Dean & Nicholas, 2018; Dean et al., 2023; Dean, Knapp, et al., 2018; Dean, Schmitz, et al., 2018; Dobkin et al., 2018; Gladstone & Whillans, 2018; Gupta et al., 2014; Hojman et al., 2016; Houle et al., 2015; Israel et al., 2014; Knapp & Dean, 2018; Lee & Song, 2015; Li et al., 2015; Meier & Sprenger, 2012; Meltzer et al., 2011; Nicholas et al., 2020). In the United States, an individual’s consumer credit history represents a cumulative series of financial decisions, based on a person’s potential to manage the timely payment schedules of loans, bills, credit cards, and debts (Israel et al., 2014; Li et al., 2015). Credit history determines one’s creditworthiness, as represented by a credit score, or ability to attract additional capital in the form of loans. The most used score in the United States is the FICO score, which ranges from 300 to 850, with greater numbers representing greater creditworthiness. Both creditworthiness and credit history are distinct from consumer debt, which is the balance of debt that one has accumulated. A person with large well-managed debt may have good credit scores, while poorly managed small amounts of debt could result in poor credit scores. Credit scores and histories determine what products and resources are available to aid in everyday life: home mortgage interest rates, auto loan, and car insurance premiums, school loans for students who take out their own debt, employment eligibility, and availability of financing for medical care (Traub, 2013).

Credit may influence health in the same way as other socioeconomic indicators, through: psychological processes,(Bartley, 2004) shaping environmental exposures (Lynch et al., 2001), reflecting engagement in risky health behaviors (Stringhini et al., 2011), and influencing self-perceptions of placement in the social hierarchy (Marmot & Wilkinson, 2005) that are reflected in somatic stress responses (Brunner & Marmot, 2005). Our recent work highlights two overarching pathways by which credit and health are linked. First, credit may be predictive, by helping us to understand who is more likely to develop, treat, or manage a condition. Second, credit is a response to the financial consequences of healthcare treatments and long-term disease impact (Dean & Nicholas, 2018).

On one hand, good premorbid consumer credit may reflect one’s ability to manage health and prevent disease. For example, about half of U.S. employers use credit histories (Traub, 2013) in their decisions to hire prospective employees (Kiviat, 2019; O’Brien & Kiviat, 2018), which in turn influence one’s income, retirement investments, and access to employer-sponsored health insurance (Board of Governors of the Federal Reserve System, 2015). Older adults may or may not still be in the workforce, but their credit may have influenced their prior ability to get jobs, subsequent income, and the retirement savings available to manage debts. In influencing one’s job, it may also influence their health through the risk of occupational hazards or physical toll a job may take. Similarly, attending school may require loans, the terms of which can be dictated by credit, and purchasing or renting a home is often dependent on the ability to secure credit to do so (Nelson, 2010; O’Brien & Kiviat, 2018). In shaping where people work and live, credit shapes environmental exposures and resources for managing health and disease.

It also may be an early marker of those who are at high-risk for disease onset: changes in one’s ability to manage their financial affairs may reflect their mental or physical state, and poorly managed debt and finances will be then reflected in their credit score. Changes in credit score may even reflect sub-threshold changes in health that lead up to a major health diagnosis (Dean et al., 2023). For example, if a patient incurs debt to pay for healthcare utilization to care for symptoms of a disease before being diagnosed with a disease (e.g., care for yeast infections or blurred vision prior to a diabetes diagnosis), and is unable to manage that debt well.

On the other hand, when a patient gets sick and needs to use healthcare services, they may incur debt which, if ill-managed, may influence one’s creditworthiness. This may be the case for acute physical ailments that require care, but not necessarily for general health declines that do not require acute care that would incur costs. An analysis by Dobkin found that even 1 hospitalization for an acute condition lowered credit limits and scores: 4 years following hospitalization, credit scores had declined and credit limits had declined by 5.5% (~$2,215 in their sample; Dobkin et al., 2018).

With few exceptions, the body of literature on consumer credit has generally only assessed credit changes in response to a disease (postmorbid) than before a disease (premorbid). Studies assessing both pre and postmorbid credit have focused on changes in cognition. For example, one study of older U.S. adult Medicare beneficiaries found a greater risk of missing payments on credit accounts up to 6 years prior to an Alzheimer’s Disease and Related Dementias (ADRD) diagnosis and a greater likelihood of missing payments up 3 months after diagnosis when compared to those who did not develop ADRD (Nicholas et al., 2020). Our own analysis of The Advanced Cognitive Training for Independent and Vital Elderly (ACTIVE) data showed that changes in credit may begin even earlier prior to an ADRD diagnosis, with associations before and after the prodromal phase of ADRD known as mild cognitive impairment (MCI). We found that each 50-point increase in credit score was associated with up to 8% lower odds of MCI in the next 3 years, while having new unpaid collections over doubled the odds of having MCI in the next 3 years. MCI was also associated with subsequent credit score declines and a 47%–71% greater risk of having a new unpaid collection in the next 4 years (Dean et al., 2023).

The ACTIVE study data offers a unique opportunity to assess the temporality of changes in credit and health among a cohort of older adults, across a range of physical and mental health outcomes over a 20-year period. Older adults are important to study with regard to credit, given that due to the erosion of social safety nets in the US, older adults are increasingly at risk of default and bankruptcy (Thorne et al., 2019). To the extent that we are aware, and after an exhaustive review of both domestic and international studies, this study will be the first opportunity to examine the timing between credit score changes and a general set of self-reported health outcomes. This analysis aims to assess the temporal ordering of credit and health changes by addressing the questions: (a) Can credit scores or changes in credit scores predict self-reported general health? and (b) How quickly might changes in health influence subsequent credit scores?

We hypothesized that poor credit history is associated with future poor physical and mental health and that having poor physical and mental health is associated with a subsequently poor credit score. Additionally, we explored the degree to which changes in credit scores predict upcoming health outcomes, and whether general health outcomes predict changes in future credit characteristics. The results of this analysis will provide evidence for whether credit may be an indicator for future physical and mental health functioning and illustrate which diseases might be best predicted or influenced by consumer credit score changes.

## Research Design and Methods

### Sample

ACTIVE was a four-arm, multi-site, single-blind, randomized controlled trial (*N* = 2,802) with recruitment from March 1998 to October 1999 in six metropolitan areas, designed to study the effectiveness of three cognitive training interventions compared to a no-contact control arm (Ball et al., 2002; Jobe et al., 2001). Participants had to be >65 years of age, which would make the vast majority eligible for health insurance under Medicare, the US’s health insurance program for older adults. Exclusion criteria included: significant cognitive dysfunction (Mini-Mental State Examination [MMSE] score <23; Folstein et al., 1975); functional impairment (dependency or regular assistance in activities of daily living (ADL) as measured by the Minimum Dataset (MDS) Home Care (Morris & Morris, 1997); self-reported diagnosis of Alzheimer’s disease; stroke within the last 12 months; certain cancers; current chemotherapy or radiation therapy; or poor vision, hearing, or communicative ability that would have interfered with the interventions or outcome assessments). Each treatment arm received ten 60–75-min sessions of training in small groups over 5–6 weeks for memory, speed of processing, or reasoning. Intervention details have been previously described (Ball et al., 2002; Jobe et al., 2001).

The ACTIVE study was approved by institutional review boards at the University of Alabama at Birmingham, Indiana University, Johns Hopkins University in Baltimore, Pennsylvania State University, and Wayne State University. For this project, we linked the survey data to credit data provided by TransUnion, a global information solutions company. The ACTIVE team sent TransUnion a dataset with minimally necessary identifiable information that enabled TransUnion to append credit history data to the ACTIVE dataset. After appending the data, TransUnion removed the original identifiers and sent the ACTIVE team a de-identified dataset with new unique study IDs, so that the original participants could not be re-identified.

The analysis sample consisted of ACTIVE participants who had matched credit data in 2001. ACTIVE follow-up interviews were conducted 1, 2, 3, and 5 years after baseline. Credit scores were matched to each of these follow-up visits using the closest year before or after the visit (January 2001, 2002, 2003, 2004, 2005, 2007, 2009, 2010, 2011, 2014, 2017), based on name, address, and Social Security Number provided at the ACTIVE baseline interview. (See Supplementary Figure 1 for a timeline and sources of data collected). Credit history may be missing due to lack of available identifying data (e.g., address) that allowed for matching to ACTIVE data.

### Measures

Health was assessed using the SF-36 self-reported general health scale (Ware Jr & Sherbourne, 1992). The SF-36 is a validated scale measuring domains of self-reported physical and mental health and functioning, giving an overall score that combines two subdomains of physical and mental health composite scores. We assessed three different health outcomes: (1) a dichotomous outcome of onset of poor general health (defined as an SF-36 with a *z*-score of <50, representing having poorer health than half the standard population), where higher values represented the onset of poor health; (2) a physical; and (3) mental health continuous composite scores, where higher values represented better physical or mental health, respectively.

We separately assessed four consumer credit history variables: continuous credit score, and prevalence of major derogatory accounts, and prevalence of any unpaid accounts in collections, with and without medical debt. *Consumer credit score* was represented by the VantageScore® 2.0, a proprietary method that the three major credit bureaus in the United States use as a summary measure of the credit risk of an individual based on past credit events. Scores range from 501 to 990, with higher numbers reflecting higher creditworthiness. Consistent with previous studies (Dean, Knapp, et al., 2018; Knapp & Dean, 2018), we rescaled the credit score into 10 categories of 50-point increments for interpretability of results. To assess short-term defaults, we assessed *major derogatory accounts* within each time period (none vs any), reflecting accounts of any amount that are not paid within 120–180 days following a due date; this measure suggests a likelihood of default (collections, charge-offs, foreclosures, bankruptcies, and repossessions) and would have negative implications on a credit score. We assessed *any unpaid collections accounts* (none vs any), which are accounts that go unpaid for 180 days beyond their due debt and whose debt is sold to a third party (charge-off). Paying off a collection might not improve credit score since even paid collections are still considered a derogatory mark. We assessed any unpaid collections accounts *including* medical debt and *excluding medical debt*. For each of the defaulted accounts, we assessed both the history (prevalence) of defaults and new (incident) defaults for each time period. We excluded delinquent accounts, which would be accounts that are unpaid for as little as 1 day past the due date and might instead reflect administrative challenges with payment (payment posting too late despite attempts to pay near the due date), rather than actual negligence. Credit history variables captured a look-back period that summarized repayment behavior for up to 36 months. We used a last value carried forward for missing credit data, due to an inability to match the participant’s address at that time point.

We included covariates from both the ACTIVE surveys and the credit reports. From the survey, we included: the group (dummy codes for each of the four groups), baseline age, sex, self-reported race/ethnicity, and education. From the credit reports, we included current home or auto loan status (may reflect the use of credit due to ongoing loans as opposed to new uses of credit). In the models regressing credit history on poor health, we additionally controlled for past credit history.

### Analysis

We calculated descriptive statistics of demographics, SF-36 domains, and credit score variables across study visits. Means and standard deviations were reported for continuous variables. Frequencies and percentages were used to summarize categorical variables. Chi-square tests were used to assess the difference in demographic characteristics and self-rated health outcomes at baseline between the matched and unmatched samples. To assess the time effect on consumer credit and determine if this association differed among those with and without poor health, we regressed consumer credit on time, for participants with or without poor health, and with an interaction term for time and health status. To illustrate the fluctuation of consumer credit before and after onset of poor health, we plotted annual credit scores each year for ACTIVE participants for those who maintained good health, for those who ever reported poor health, and before and after the year of poor health onset.

We used a series of time-lagged regression models to assess the temporal associations between consumer credit and poor health. To account for the correlation between multiple visits for an individual, we used Generalized Estimating Equations (GEE) for all models (Liang & Zeger, 1986). For our first hypothesis, the credit variable at the previous visit was the exposure, and subsequent poor health was the dichotomized outcome in each time-lagged model. Odds ratios (OR) and their 95% confidence intervals (CI) from GEEs with logit link were reported as the effect of credit history on poor health at the follow-up visits. For assessing whether changes in credit preceded poor health, we calculated how much change in credit there had been since the previous visit (e.g., previous visit credit score minus current visit credit score); positive coefficients represent score losses.

For the second hypothesis, poor health was the exposure, and consumer credit was the outcome variable at subsequent visits. For assessing whether changes in consumer credit followed onset of poor health, we calculated the degree to which credit variables changed from one visit to the next upcoming 4 years of visit (e.g., credit score at current visit minus credit score at next visit) and whether the participant had new defaults (derogatory accounts or unpaid accounts in collections), adjusting for baseline credit score. We also examined whether the type of collection mattered: medical versus. non-medical. GEE models with identity link were used to assess the relationship between and the credit score at follow-up visits. The coefficients and their corresponding 95% confidence intervals were reported. For dichotomized outcomes, ORs (95% CIs) were reported.

We performed supplemental analyses on continuous composite scores for the SF-36 physical health and mental health domains. All analyses were performed using SAS (v.9.4; SAS Institute Inc, Cary, North Carolina, USA).

## Results

**Figure 1.**
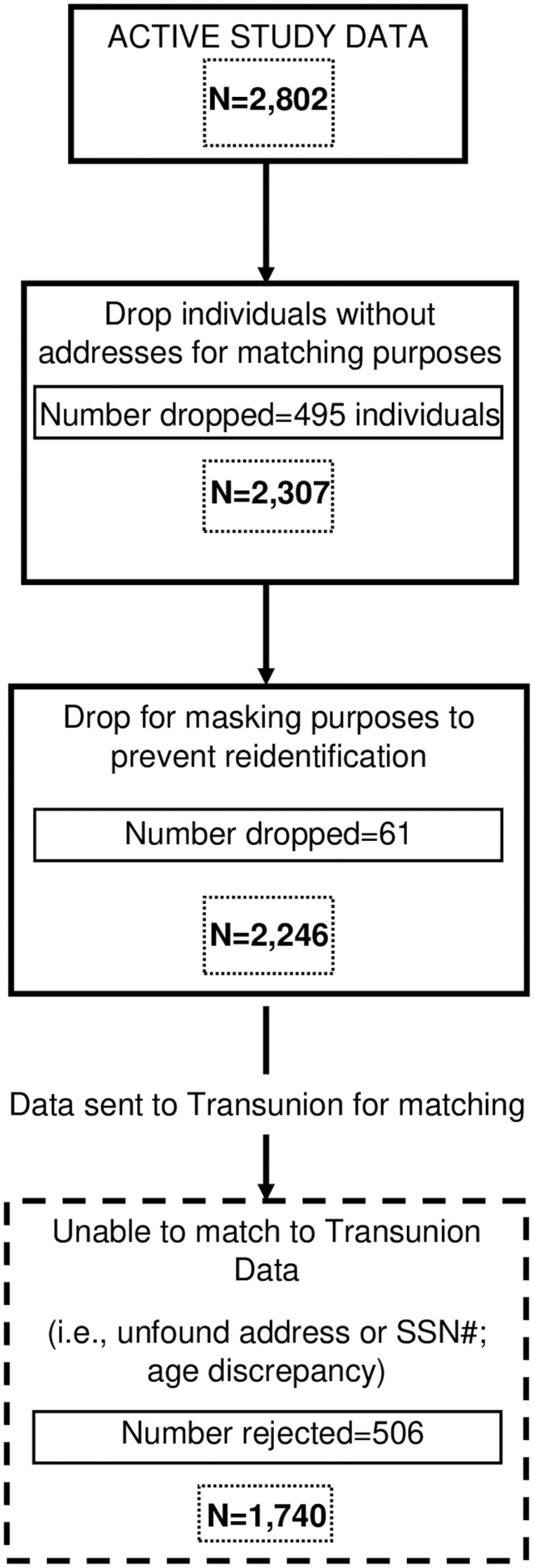
ACTIVE-TransUnion match process diagram. ACTIVE = Advanced Cognitive Training for Independent and Vital Elderly study.

Of the 2,802 ACTIVE study participants, 1,740 were included in our final analytic sample (Figure 1) after excluding participants due to a lack of data enabling us to match to TransUnion data or due to unique characteristics of study participants that could have enabled re-identification. The matched sample was demographically similar to the full ACTIVE sample, with approximately two-thirds of the sample under age 75, most being White (71%) and female (76%). Most did not have a currently reported auto or home loan (62%). Up to one-fourth of participants had a major derogatory, unpaid, or collections account in any given year (Table 1).

**Table 1.** Demographic Characteristics of ACTIVE Study Participants Matched to TransUnion Credit Score Data (*N* = 1,740)

Variable	Year 1 (Visits 2 & 3)	Year 2 (Visit 4)	Year 3 (Visit 5)	Year 5 (Visit 6)
Demographic characteristics, *N*	1,740	1,733	1,727	1,720
Age Group, *n* (%)				
60–69	570 (33%)			
70–74	572 (33%)			
75–79	366 (21%)			
80+	232 (13%)			
Race, *n* (%)				
Black, including Hispanic	507 (29%)			
White, including Hispanic	1,233 (71%)			
Sex, *n* (%)				
Female	1,331 (76%)			
Male	409 (24%)			
Education, *n* (%)				
≤12 years of education	697 (40%)			
13–16 years of education	577 (33%)			
>16 years of education	466 (27%)			
Current home or auto loan	666 (38%)	661 (38%)	654(38%)	636 (37%)
Self-rated health outcome, *n*	1,464	1,446	1,387	1,247
Poor health (SF-36 < 50), *n* (%)	167 (11%)	184 (13%)	176 (13%)	160 (13%)
Good health (SF-36 ≥ 50), *n* (%)	1,297 (89%)	1,262 (87%)	1,211 (87%)	1,087 (87%)
Credit variables				
All study participants, *N*	1,740	1,733	1,727	1,720
VantageScore (same year), m (SD)	818 (93)	820 (97)	823 (100)	824 (99)
Subprime (≤700), *n* (%)	229 (13%)	246 (14%)	247 (15%)	235 (14%)
Near Prime (701-800), *n* (%)	340 (20%)	308 (18%)	281 (17%)	288 (17%)
Prime (>800), *n* (%)	1,157 (67%)	1,154 (68%)	1,164 (69%)	1,150 (69%)
Any major derogatory versus none, *n* (%)	389 (22%)	413 (24%)	441 (26%)	445 (26%)
Any unpaid collection versus none, *n* (%)	167 (10%)	186 (11%)	205 (12%)	214 (12%)
Any collections, excluding medical, *n* (%)	148 (9%)	161 (9%)	175 (10%)	186 (11%)
Among those with poor self-rated health, *n*	167	184	176	160
VantageScore (same year), m (*SD*)	804 (99)	804 (108)	821 (99)	806 (102)
Subprime (≤700), *n* (%)	21 (13%)	40 (22%)	26 (15%)	29 (18%)
Near Prime (701–800), *n* (%)	47 (28%)	29 (16%)	27 (15%)	34 (22%)
Prime (>800), *n* (%)	98 (59%)	114 (62%)	122 (70%)	94 (60%)
Any major derogatory versus none, *n* (%)	48 (29%)	55 (30%)	48 (27%)	48 (30%)
Any unpaid collection versus none, *n* (%)	25 (15%)	29 (16%)	27 (15%)	26 (16%)
Any collections, excluding medical, *n* (%)	21 (13%)	26 (14%)	18 (10%)	24 (15%)
Among those with good self-rated health, *n*	1,297	1,262	1,211	1,087
VantageScore (same year), m (*SD*)	824 (91)	827 (91)	827 (97)	833 (93)
Subprime (≤700), *n* (%)	156 (12%)	150 (12%)	160 (13%)	135 (13%)
Near prime (701–800), *n* (%)	238 (18%)	227 (18%)	200 (17%)	169 (16%)
Prime (>800), *n* (%)	899 (70%)	872 (70%)	837 (70%)	773 (72%)
Any major derogatory versus none, *n* (%)	260 (20%)	260 (21%)	278 (23%)	240 (22%)
Any unpaid collection versus none, *n* (%)	104 (8%)	111 (9%)	124 (10%)	110 (10%)
Any collections, excluding medical, *n* (%)	95 (7%)	94 (8%)	108 (9%)	92 (9%)

*Notes*: ACTIVE = Advanced Cognitive Training for Independent and Vital Elderly study; SD = standard deviation; SF = short form.

Among the subset reporting SF-36 in any given year, up to 13% self-reported poor health. There was no difference in the proportion of people with poor health between the matched and unmatched groups (Supplementary Materials). Those that were unable to be matched to credit data were older and had a higher proportion with education <12 years, compared to those in the matched group.

**Figure 2.**
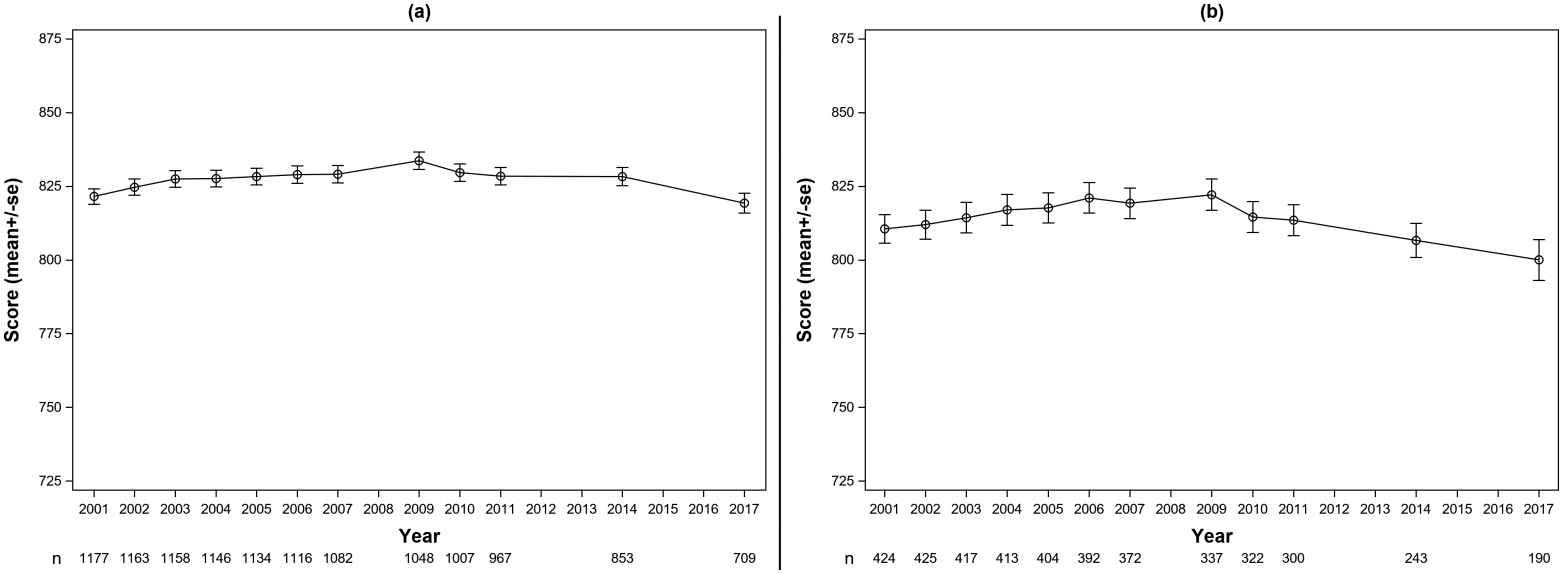
Plot of mean consumer credit score over time during 2001–2017 for participants who (a) never reported poor self-rated health (SF-36 ≥ 50); (b) ever reported poor self-rated health (SF-36 < 50). SF = short form.

**Figure 3.**
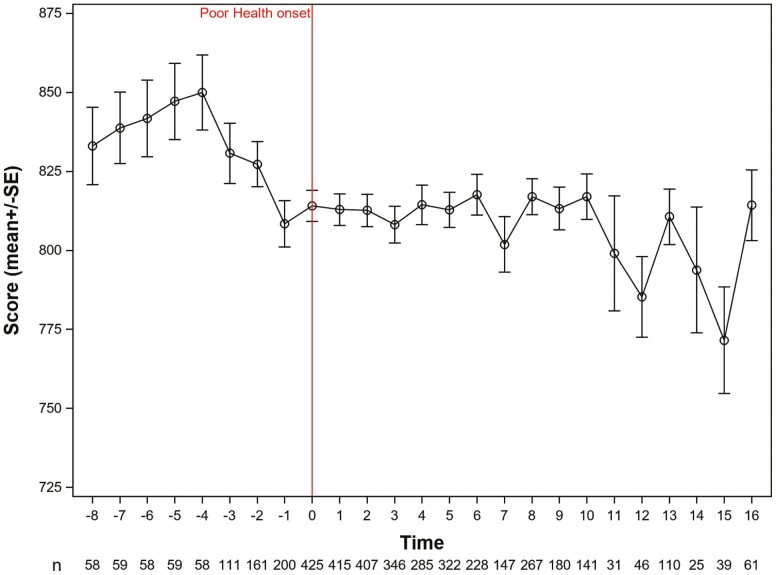
Plot of mean consumer credit score over time based on timing of poor health onset (SF-36 < 50). SF = short form.

The plots in Figure 2 show the trajectories of credit scores for those who never had poor health (2a) and whoever had (2b) poor health onset during 2001–2017. In 2001, when the cohort started, the mean credit score of people who would never have poor health was 822, while the mean credit score of people who eventually had poor health was 811. The credit scores of those with poor health were significantly lower at baseline than the credit scores of those without poor health (β = −0.26; *p* = .018). Overall, there was a significant decrease in credit scores over time for both those without poor health (β = −0.017) and for those with poor health (β = −0.040; *p* = .007). For those with poor health onset (Figure 3), we show the event-study graph, plotting credit scores before and after the poor health onset (time 0). Credit scores for people with poor health began to decline approximately 4 years prior to poor health onset and remain at a slightly lower level than those without poor health for the remainder of the time period.

Associations between up to 3 years prior credit history and future risk of poor self-rated health are shown in Table 2. Each 50-point increase in credit score was associated with a trend toward a lower risk of reporting poor overall health: marginally significant findings of 5% lower odds of in the next 1 year, a 7% lower odds in the next 2 years, and a statistically significant finding of 13% lower by 3 years. Better prior credit scores were associated with better physical health (Supplementary Table 2) only at 3 years later (β = 0.91 [0.02, 1.81]) but associated with mental health (Supplementary Table 3) each year for up to 3 years (β = 0.96 [0.49, 1.44] in year 1; β = 1.16 [0.61, 1.71] in year 2; and β = 1.37 [0.68, 2.07] in year 3). A drop in credit score was associated with a 10% greater odds of reporting overall poor health in the next 1 year, but not for physical and mental health subdomains. Having a major derogatory mark on one’s account was associated with a 40% greater odds of overall poor health in the next year, and an 86% in the next 3 years. Having a major derogatory mark was associated with worse physical and mental health for up to 3 years later. For physical health, associations showed a gradient over time, with the strongest signals showing 3 years prior to poor physical health onset (β = −6.62 [−10.6, −2.64]), and waning closer to the time of poor physical health onset (β = −4.44 [−7.42, −1.46] in year 2 and β = −3.20 [−5.67, −0.73] in year 1). Similarly, for mental health, associations showed a gradient over time with the strongest signals 3 years prior to poor mental health onset (β = −4.83 [−7.93, −1.74]) and waning closer to the time of poor mental health onset (β = −3.10 [−5.67, −0.53] in year 2 and β = −2.02 [−4.10, 0.07] in year 1). Having any unpaid collections (which can include medical debt) was associated with 57% greater odds of overall poor health in the next 1 year, and associated with worse physical health subdomains scores at 2 years (β = −5.28 [−9.35, −1.23]) and 3 years (β = −6.43 [−12.1, −0.79]) later, and worse mental health at 1 year later (β = −4.65 [−7.52, −1.79]). Having existing non-medical debt in collections was not associated with upcoming overall poor health, but was associated with worse physical health at 1 year (β = −3.87 [−7.40, −0.34]) and worse mental health for up to 3 years (β = −4.19 [−7.15, −1.24] in year 1; β = −3.68 [−7.35, −0.01] in year 2; and β = −5.51 [−10.0, −0.98] in year 3).

**Table 2.** Odds of Poor Self-Rated Health Based on Past Credit History: Results From ACTIVE Study Participants Matched to TransUnion Credit Score Data

Variable	Odds of poor health (SF-36 < 50)
	β (95% CI)
Credit scores	
Score as continuous 1 year prior	0.95 (0.89, 1.01)
Score as continuous 2 years prior	0.93 (0.87, 1.00)
Score as continuous 3 years prior#	0.87 (0.80, 0.96)^*^
Loss in credit score since the previous visit	
Score loss from 1 year prior	1.10 (1.02, 1.18)^*^
Score loss from 2 years prior	1.06 (0.96, 1.18)
Score loss from 3 years prior	1.09 (0.93, 1.26)
Presence of derogatory account	
Major derogatory 1 year prior	1.40 (1.08, 1.82)^*^
Major derogatory 2 years prior	1.33 (0.97, 1.82)
Major derogatory 3 years prior#	1.86 (1.24, 2.79)^*^
Presence of unpaid collections accounts	
Any unpaid collections 1 year prior	1.57 (1.15, 2.13)^*^
Any unpaid collections 2 years prior	1.34 (0.87, 2.05)
Any unpaid collections 3 years prior#	1.65 (0.95,2.85)
Presence of account in collections, excluding medical debt	
Any collection excluding medical debt 1 year prior	1.38 (0.97, 1.95)
Any collection excluding medical debt 2 years prior	0.84 (0.52, 1.35)
Any collection excluding medical debt 3 years prior#	1.32 (0.73, 2.37)

*Notes*: Credit scores are treated as continuous in 50-point increments. Covariates included are ACTIVE group, baseline age, sex, race/ethnicity, education, and current asset ownership status; models where a change in credit history was the predictor included previous time point’s credit as a covariate. Major derogatory accounts are those unpaid for up to 180 days; collections accounts are those unpaid for longer than 180 days and whose debt is sold to a third party (charge-off). ACTIVE = Advanced Cognitive Training for Independent and Vital Elderly study; CI = confidence interval; SF = short form.

^*^ *p* < .05.

Associations between the postmorbid consumer credit and poor health can be found in Table 3. Up to 3 years after poor health onset, credit scores continued to see significant decrements with larger decrements as time increased (β=−0.08 [−0.15, −0.02] in year 1; β=−0.11 [−0.21, −0.01] in year 2, and β=−0.16 [−0.28, −0.04] in year 3). Better physical health (Supplementary Table 4) was associated with future higher credit scores up to 3 years later (β = 0.001 [0.000, 0.002] in year 1; β = 0.002 [0.001, 0.004] in year 2, and β = 0.002 [0.000, 0.004] in year 3). Mental health (Supplementary Table 5) was associated with future higher credit scores for up to 2 years later (β = 0.001 [0.001, 0.002] in year 1 and 0.003 [0.001, 0.004] in year 2) and 4 years later (β = 0.002 [0.000, 0.005]).In each of the 3 years after the onset of overall poor health, the degree of loss in credit score was significant (β = 0.08 [0.02, 0.15] in year 1; β = 0.11 [0.01, 0.21] in year 2, and β = 0.16 [0.04, 0.28] in year 3). Better physical health was associated with less credit score losses at up to 2 years (β=−0.001 [−0.002, −0.000] in year 1 and β=−0.002 [−0.003, −0.001] in year 2), and better mental health was associated with less credit score loss for up to 4 years (β=−0.002 [−0.002, −0.001] in year 1; β=−0.003 [−0.004, −0.001] in year 2, β=−0.002 [−0.004, −0.000] in year 3, and β=−0.003 [−0.004, −0.000] in year 4). Among major defaults, in the form of derogatory accounts, new accounts, and unpaid collections, only new accounts (excluding medical debt) going into collections was statistically significant at 2 years after overall poor health onset (OR = 1.97 [95% CI: 1.27, 3.06]) and was not associated with physical or mental health subdomains.

**Table 3:** Regression Results for Past Poor Self-Rated Health (SF-36 < 50) on Future Consumer Credit: Results From ACTIVE Study Participants Matched to TransUnion Credit Score Data

Variable	β	OR	(95% CI)
Credit score			
Score as continuous 1 year after	−0.08		(−0.15, −0.02)^*^
Score as continuous 2 years after	−0.11		(−0.21, −0.01)^*^
Score as continuous 3 years after	−0.16		(−0.28, −0.04)^*^
Score as continuous 4 years after	0.001		(−0.14, 0.14)
Credit score loss at the upcoming visits			
Score loss 1 year after	0.08		(0.02, 0.15)^*^
Score loss 2 years after	0.11		(0.01, 0.21)^*^
Score loss 3 years after	0.16		(0.04, 0.28)^*^
Score loss 4 years after	0.001		(−0.14,0.14)
Report of new derogatory account at the upcoming visit			
Major derogatory 1 year after		1.25	(0.82, 1.92)
Major derogatory 2 years after		1.14	(0.80, 1.63)
Major derogatory 3 years after		1.10	(0.78, 1.56)
Major derogatory 4 years after		1.19	(0.82, 1.74)
Report of new unpaid collections at upcoming visit			
Any unpaid collections 1 year after		1.50	(0.94, 2.41)
Any unpaid collections 2 years after		1.37	(0.89, 2.11)
Any unpaid collections 3 years after		1.33	(0.89, 2.00)
Any unpaid collections 4 years after		1.02	(0.63, 1.64)
Report of new accounts in collections, excluding medical debt, at upcoming visit			
Any collection excluding medical debt 1 year after		1.61	(0.94, 2.73)
Any collection excluding medical debt 2 years after		1.97	(1.27, 3.06)^*^
Any collection excluding medical debt 3 years after		1.23	(0.78, 1.95)
Any collection excluding medical debt 4 years after		1.18	(0.73, 1.91)

*Notes*: Credit scores are treated as continuous, in 50-point increments. Covariates included are ACTIVE group, past credit score, baseline age, sex, race/ethnicity, education, and current asset ownership status. Major derogatory accounts are those unpaid for up to 180 days; collections accounts are those unpaid for longer than 180 days and whose debt is sold to a third party (charge-off). ACTIVE = Advanced Cognitive Training for Independent and Vital Elderly study; CI = confidence interval; OR = odds ratio.

^*^ *p* < .05.

## Discussion and Implications

Our goal was to examine the associations between pre and postmorbid consumer credit and the onset of self-reported poor health among older adults in the ACTIVE study. Our results suggest that poor credit scores may be signals of future poor health in the coming 3 years, and that after poor health onset, credit scores may be impacted for up to another 3 years. Credit losses showed in the one year prior to poor health onset, and up to 3 years after poor health onset. Other indicators of credit were less consistently linked either before or after poor health. Supplemental analyses suggest that credit may have a larger impact on future mental than physical health; poor mental health can lead to losses in future credit up to 4 years later, while poor physical health can lead to losses in future credit up to 2 years later. Our findings advance the literature on understanding the temporal associations between credit, and the possible mechanisms linking credit to health, which may differ pre and postpoor health onset. Findings purport two potential pathways linking credit and health: (a) credit may be predictive of health that is declining and (b) poor credit may be a consequence of the ability to manage the financial impacts of healthcare treatments due to poor health (Dean & Nicholas, 2018).

Our results pointed to a possible trend of poor credit leading up to poor health onset, with the strongest signal being 3 years prior to onset and weaker signals closer to the time of poor health onset. Both physical and mental health subscore domains were also most strongly associated with credit 3 years prior. Similarly, our findings in MCI (Dean et al., 2023) found the strongest signals between credit scores and upcoming health at further time points away than closer time points. It is possible that we see strongest signals at 3 years, rather than 2 or 1 year prior to health onset because family members or others may be intervening after precipitous drops or changes in credit score even before a person is self-reporting poor health (National Institute on Aging, 2017). That is to say, it is possible that credit scores were even poorer more than 3 years out, as studies show that there may be up to a 6 or more year lead time, and that scores closest to the time of recognized poor health may reflect credit that has already been intervened upon before it could get worse. The presence of some sort of intervention may also be supported by our findings in unpaid collections, which suggest that medical debt may be most associated with defaults in the year closest to poor health onset. Further bolstering this point are the physical and mental health subscores: unpaid collections including medical debt is associated with poor physical health 2 and 3 years later, and with poor mental health only in 1 year later. Changes in physical health may be more noticeable early on and subject to intervention earlier than mental health changes.

While premorbid credit shows weak signals, there are stronger associations and consistent findings for worse postmorbid credit after the onset of poor health. Better physical and mental health is associated with subsequent better credit scores and lower likelihood of credit losses. After the onset of poor health, drops in credit scores are significant for up to 3 years, as are significant losses in credit; however, this does not seem to significantly impact the likelihood of major defaults in a consistent way. This may be because unlike high-cost conditions like cancer, which are known to cause major defaults and bankruptcy (Ramsey et al., 2013, 2016), general health changes may not incur high enough medical costs such that it would turn into difficult-to-manage debt. Especially in the case of a group of older adults insured under Medicare, they may be more insulated from the economic ramifications of large health shocks. Even so, even small amounts of poorly managed debt could still negatively impact credit. It may also be that patients may be leveraging existing equity or credit to pay for medical expenses, thus avoiding the risk of missed repayment or credit defaults. In that sense, even though credit scores are decreasing, it may be to avoid the even more detrimental impacts of accounts going into collections. Similar to our conclusions for premorbid credit, postmorbid credit’s association with general self-reported health may be different than the pathways through which credit is associated with diagnosed physical and mental health outcomes.

Our findings may have implications for the early detection of future poor mental or physical health. Particularly, having major credit defaults in older age could be a potential trigger for intervention or signal a heightened need for screening and monitoring of changes in health. Given that the focus of our analysis is on subjective changes in self-rated health, which may be a precursor to detectable changes in objective health, credit changes for older adults may be a very early signal of health changes to come in the next three years. Monitoring credit changes for older adults could allow for earlier detection and intervention of both physical and mental ailments. After poor health onset, credit might be monitored to assess the financial well-being of an older adult, who due to poor health onset, may be susceptible to future health declines and ongoing health care needs. Especially given older adults’ increased risk of unmanageable healthcare costs (Thorne et al., 2019), they may need assistance to ensure that their credit products are available to leverage, to be in the best position to preserve their health and longevity. Prospective studies of interventions that use credit as an early marker for intervention, or that examine how stabilizing credit after poor health onset should be considered.

### Limitations

Limitations of our analysis suggest that our findings may overestimate or underestimate the associations between credit and poor self-rated health. The ACTIVE sample of relatively healthy, community-dwelling older adults, the majority of whom were healthy at baseline and had high credit scores, limiting generalizability to the population of older adults living with certain chronic conditions. Yet, working with a sample of relatively healthy adults helped us to make stronger inferences about how credit changes in response to a downturn in health. Nearly one-third of the ACTIVE sample was unable to be matched to credit data, mostly due to challenges with identifying an address for the participant. Inconsistent address information may be a marker of residential instability that may be related to consumer credit, meaning we may be missing a sample of people who are the most impaired and underestimate our associations; however, we did not find differences in health when we compared the matched to unmatched groups (see Supplementary Table 1). Those with unmatched credit data were older and had fewer years of education which might suggest that they had higher or lower credit scores than the matched sample. Results from the matched sample may be biased based on demographic group differences, but the direction and degree of that bias is unknown. Results from the matched results may be biased, but the direction of that bias is unknown. SF-36 self-rated health is a general health outcome, and unlike cognition outcomes explored in other studies, does not give a sense of what types of mechanisms may be at work between credit and poor health onset; however, our supplemental analysis of the physical and mental health composite scores suggest that mental health may be more strongly associated with pre and postmorbid credit than physical health.

It is possible that credit data reflected the activities of the financial manager of the household, who may not have been the ACTIVE participant for whom health was assessed. The study does not have information on the financial manager; however, this likely means our study underestimates the relationship between health and credit, as some financial challenges may have been avoided due to intervention by a financial manager. In fact, the intervention of a financial manager may largely explain our findings for premorbid credit history.

## Conclusion

Our results suggest that consumer credit scores and defaults, may predict upcoming poor health up to 3 years later, and that a downturn in health can have impacts on credit scores, but not credit defaults, for 3 years afterward. The magnitude of these pre and postmorbid associations are strongest at the 3-year mark. Having a major derogatory account or having a sudden loss in credit may be a time to especially monitor older adults for changes in health, and consider opportunities for more intensive screening or early health intervention. After a downturn in health, supporting older adults to manage their overall debt may help stabilize their credit. Future prospective studies should directly assess whether monitoring credit before or after health changes in older adults could be beneficial.

## Supplementary Material

igae016_suppl_Supplementary_Material

## Data Availability

ACTIVE data and documentation from 1998 to 2010 are available via the National Archive of Computerized Data on Aging. Credit score data are proprietary and provided by TransUnion©. This study was not preregistered.
