# Supplementary material for: Does Consumer Credit Precede or Follow Health Among Older Adults? An Investigation in the Advanced Cognitive Training for Independent and Vital Elderly (ACTIVE) Trial
Source: Innov Aging. 2024 Feb 22;8(3):igae016. doi: 10.1093/geroni/igae016 (PMC10953618; doi:10.1093/geroni/igae016)
Supplement: igae016_suppl_Supplementary_Material [file igae016_suppl_supplementary_material.docx]

*Innovation in Aging* Supplementary Material: Dean, Lorraine; Chung, Shang-En; Gross, Alden L.; Clay, Olivio J.; Willis, Sherry L.; McDonough, Ian M.; Thomas, Kelsey R.; Marsiske, Michael; Aysola, Jaya; Thorpe, Roland J.; Felix, Cynthia; Berkowitz, Melissa; Coe, Norma B. Does consumer credit precede or follow health among older adults? An investigation in the Advanced Cognitive Training for Independent and Vital Elderly (ACTIVE) Trial.

Supplemental Figure 1. Timeline of ACTIVE and Credit History Data Collection


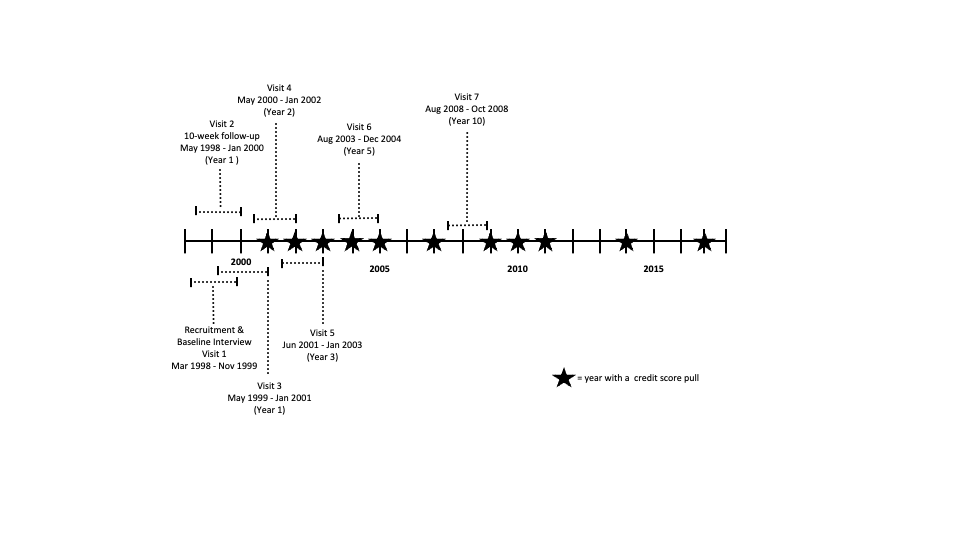


Supplemental Table 1: Comparison between participants who were matched or unmatched to credit score data

| N (%) for categorical variables and mean(sd) for continuous variables | Matched group  (N=1,740) | Unmatched  Group  (n=506) | p-value from $\boldsymbol{\chi}^{\boldsymbol{2}}$ |
| --- | --- | --- | --- |
|  |  |  |  |
| Poor self-rated health | 167 (11%) | 56 (12%) | 0.666 |
| Age Group |  |  | <.001 |
| 65-69 | 570 (33%) | 134 (26%) |  |
| 70-74 | 572 (33%) | 137 (27%) |  |
| 75-79 | 366 (21%) | 130 (26%) |  |
| 80+ | 232 (13%) | 105 (21%) |  |
| Race |  |  | 0.701 |
| Black, including Hispanic | 507 (29%) | 143 (28%) |  |
| White, including Hispanic | 1,233 (71%) | 363 (72%) |  |
| Sex |  |  | 0.459 |
| Female | 1,331 (76%) | 395 (78%) |  |
| Male | 409 (24%) | 111 (22%) |  |
| Education |  |  | <.001 |
| <=12 years of education | 697 (40%) | 254 (50%) |  |
| 13-16 years of education | 577 (33%) | 154 (30%) |  |
| >16 years of education | 466 (27%) | 98 (19%) |  |
|  |  |  |  |

Supplemental Table 2: Regression results for **composite physical health** score on past credit history (β, 95%CI): Results from ACTIVE Study Participants Matched to TransUnion Credit Score Data

|  | β (95% CI) |
| --- | --- |
|  | GEE or  Multivariate regression (#) |
| Credit scores |  |
| Score as continuous 1 year prior | 0.44 (-0.13,1.00) |
| Score as continuous 2 years prior | 0.57 (-0.12,1.25) |
| Score as continuous 3 years prior# | 0.91 (0.02,1.81)* |
|  |  |
| Loss in credit score since previous visit |  |
| Score loss from 1 year prior | -0.16 (-0.82,0.49) |
| Score loss from 2 years prior | -0.62 (-1.44,0.21) |
| Score loss from 3 years prior | -0.40 (-1.79,0.99) |
|  |  |
| Presence of derogatory account |  |
| Major derogatory 1 year prior | -3.20 (-5.67,-0.73)* |
| Major derogatory 2 years prior | -4.44 (-7.42,-1.46)* |
| Major derogatory 3 years prior# | -6.62 (-10.6,-2.64)* |
|  |  |
| Presence of unpaid collections accounts |  |
| Any unpaid collections 1 year prior | -1.97 (-5.02,1.08) |
| Any unpaid collections 2 years prior | -5.28 (-9.35,-1.23)* |
| Any unpaid collections 3 years prior# | -6.43 (-12.1,-0.79)* |
|  |  |
| Presence of account in collections, excluding medical debt |  |
| Any collection excluding medical debt 1 year prior | -3.87 (-7.40,-0.34)* |
| Any collection excluding medical debt 2 years prior | -1.99 (-6.04,2.06) |
| Any collection excluding medical debt 3 years prior# | -3.52 (-9.36,2.32) |
|  |  |

Note. Credit scores are treated as continuous in 50-point increments. Covariates included are ACTIVE group, baseline age, sex, race/ethnicity, education and current asset ownership status; models where change in credit history was the predictor included previous time point’s credit as a covariate. Major derogatory accounts are those unpaid up to 180 days; collections accounts are those unpaid for longer than 180 days and whose debt is sold to a third party (charge-off).

*p<0.05.

Supplemental Table 3: Regression results for **composite mental health score** on past credit history (β, 95%CI): Results from ACTIVE Study Participants Matched to TransUnion Credit Score Data

|  | β (95% CI) |
| --- | --- |
|  | GEE or  Multivariate regression (#) |
| Credit scores |  |
| Score as continuous 1 year prior | 0.96 (0.49,1.44)* |
| Score as continuous 2 years prior | 1.16 (0.61,1.71)* |
| Score as continuous 3 years prior# | 1.37 (0.68,2.07)* |
|  |  |
| Loss in credit score since previous visit |  |
| Score loss from 1 year prior | -0.52 (-1.17,0.13) |
| Score loss from 2 years prior | -0.12 (-0.90,0.65) |
| Score loss from 3 years prior | -0.40 (-1.79,0.99) |
|  |  |
| Presence of derogatory account |  |
| Major derogatory 1 year prior | -2.02 (-4.10,0.07) |
| Major derogatory 2 years prior | -3.10 (-5.67,-0.53)* |
| Major derogatory 3 years prior# | -4.83 (-7.93,-1.74)* |
|  |  |
| Presence of unpaid collections accounts |  |
| Any unpaid collections 1 year prior | -4.65 (-7.52,-1.79)* |
| Any unpaid collections 2 years prior | -2.99 (-6.68,0.69) |
| Any unpaid collections 3 years prior# | -4.02 (-8.41,0.38) |
|  |  |
| Presence of account in collections, excluding medical debt |  |
| Any collection excluding medical debt 1 year prior | -4.19 (-7.15,-1.24)* |
| Any collection excluding medical debt 2 years prior | -3.68 (-7.35,-0.01)* |
| Any collection excluding medical debt 3 years prior# | -5.51 (-10.0,-0.98)* |
|  |  |

Note. Credit scores are treated as continuous in 50-point increments. Covariates included are ACTIVE group, baseline age, sex, race/ethnicity, education and current asset ownership status; models where change in credit history was the predictor included previous time point’s credit as a covariate. Major derogatory accounts are those unpaid up to 180 days; collections accounts are those unpaid for longer than 180 days and whose debt is sold to a third party (charge-off).

*p<0.05.

Supplemental Table 4: Regression results for **past** **composite physical health** score on future consumer credit (β/OR, 95%CI): Results from ACTIVE Study Participants Matched to TransUnion Credit Score Data

|  | GEE model |
| --- | --- |
|  | β (95% CI) |
| Credit score |  |
| Score as continuous 1 year after | 0.001 (0.000,0.002)* |
| Score as continuous 2 years after | 0.002 (0.001,0.003)* |
| Score as continuous 3 years after | 0.002 (0.000,0.004)* |
| Score as continuous 4 years after | 0.001 (-0.000,0.004) |
|  |  |
| Credit score loss at upcoming visits |  |
| Score change 1 year after | -0.001 (-0.002,-0.000)* |
| Score change 2 years after | -0.002 (-0.003,-0.001)* |
| Score change 3 years after | -0.001 (-0.003,0.000) |
| Score change 4 years after | -0.002 (-0.004,0.000) |
|  |  |
|  | OR (95% CI) |
| Report of new derogatory account at upcoming visit |  |
| Major derogatory 1 year after | 1.00 (0.99,1.01) |
| Major derogatory 2 years after | 1.00 (0.99,1.01) |
| Major derogatory 3 years after | 1.00 (1.00,1.01) |
| Major derogatory 4 years after | 1.00 (0.99,1.00) |
|  |  |
| Report of new unpaid collections at upcoming visit |  |
| Any unpaid collections 1 year after | 0.99 (0.99,1.00) |
| Any unpaid collections 2 years after | 0.99 (0.99,1.00) |
| Any unpaid collections 3 years after | 1.00 (0.99,1.00) |
| Any unpaid collections 4 years after | 0.99 (0.99,1.00) |
|  |  |
| Report of new accounts in collections, excluding medical debt, at upcoming visit | |
| Any collection excluding medical debt 1 year after | 0.99 (0.98,1.01) |
| Any collection excluding medical debt 2 years after | 0.99 (0.98,1.00) |
| Any collection excluding medical debt 3 years after | 1.00 (0.99,1.00) |
| Any collection excluding medical debt 4 years after | 1.00 (0.99,1.00) |
|  |  |

Note. Credit scores are treated as continuous, in 50-point increments. Covariates included are ACTIVE group, past credit score, baseline age, sex, race/ethnicity, education and current asset ownership status. Major derogatory accounts are those unpaid up to 180 days; collections accounts are those unpaid for longer than 180 days and whose debt is sold to a third party (charge-off).

*p<0.05.

Supplemental Table 5: Regression results for **past composite mental health score** on future consumer credit (β/OR, 95%CI): Results from ACTIVE Study Participants Matched to TransUnion Credit Score Data

|  | GEE model |
| --- | --- |
|  | β (95% CI) |
| Credit score |  |
| Score as continuous 1 year after | 0.001 (0.001,0.002)* |
| Score as continuous 2 years after | 0.003 (0.001,0.004)* |
| Score as continuous 3 years after | 0.001 (-0.001,0.003) |
| Score as continuous 4 years after | 0.002 (0.000,0.005)* |
|  |  |
| Credit score loss at upcoming visits |  |
| Score change 1 year after | -0.002 (-0.002,-0.001)* |
| Score change 2 years after | -0.003 (-0.004,-0.001)* |
| Score change 3 years after | -0.002 (-0.004,-0.000)* |
| Score change 4 years after | -0.003 (-0.004,-0.000)* |
|  |  |
|  | OR (95% CI) |
| Report of new derogatory account at upcoming visit |  |
| Major derogatory 1 year after | 0.99 (0.99,1.00) |
| Major derogatory 2 years after | 1.00 (0.99,1.00) |
| Major derogatory 3 years after | 0.99 (0.99,1.00) |
| Major derogatory 4 years after | 0.99 (0.99,1.00) |
|  |  |
| Report of new unpaid collections at upcoming visit |  |
| Any unpaid collections 1 year after | 0.99 (0.98,1.00) |
| Any unpaid collections 2 years after | 0.99 (0.99,1.00) |
| Any unpaid collections 3 years after | 0.99 (0.98,1.00) |
| Any unpaid collections 4 years after | 0.99 (0.99,1.00) |
|  |  |
| Report of new accounts in collections, excluding medical debt, at upcoming visit | |
| Any collection excluding medical debt 1 year after | 0.99 (0.99,1.00) |
| Any collection excluding medical debt 2 years after | 0.99 (0.99,1.00) |
| Any collection excluding medical debt 3 years after | 0.99 (0.99,1.00) |
| Any collection excluding medical debt 4 years after | 1.00 (0.99,1.01) |
|  |  |

Note. Credit scores are treated as continuous, in 50-point increments. Covariates included are ACTIVE group, past credit score, baseline age, sex, race/ethnicity, education and current asset ownership status. Major derogatory accounts are those unpaid up to 180 days; collections accounts are those unpaid for longer than 180 days and whose debt is sold to a third party (charge-off).

*p<0.05.
